# Impact of chronic dietary aflatoxin B1 intake on rumen fermentation, hepatic health, oxidative stress, inflammation, and glucose–lipid metabolism in fat-tailed ewes

**DOI:** 10.1016/j.vas.2026.100758

**Published:** 2026-07-08

**Authors:** Nasibeh Ider, Hamed Khalilvandi-Behroozyar, Abdollah Ahmadpour, Farhad Farhangpajouh, Maryam Sahebi-Ala, Yanting Chen, Morteza Hosseini Ghaffari

**Affiliations:** a Department of Animal Science, Agriculture Faculty, Urmia University, postal code: 5756151818, Post box:165, Urmia, Iran; b Higher Education Centre of Shahid Bakeri, Urmia University, Miyandoab, Iran; c Faculty of Veterinary Science, Urmia University, Urmia, Iran; d College of Animal Science and Technology, Nanjing Agricultural University, Nanjing, 210095, China; e Research Institute for Farm Animal Biology (FBN), Dummerstorf D, 18196, Germany

**Keywords:** Aflatoxin B₁, Sheep, Mycotoxin, Hepatic injury, Inflammation, Oxidative stress, Glucose metabolism

## Abstract

Chronic dietary exposure to aflatoxin B₁ (AFB₁) is a major threat to ruminant health, but its integrated effects on rumen fermentation, hepatic function, inflammatory–oxidative status, and glucose–lipid metabolism in fat-tailed sheep remain insufficiently characterized. This study evaluated the effects of high-level chronic AFB₁ exposure in twenty non-pregnant, non-lactating multiparous Ghezel ewes assigned to either a control diet or the same basal diet supplemented with AFB₁-contaminated rice culture providing 500 µg AFB₁/kg dry matter for 40 d. Individual dry matter intake was not measured; however, all animals consumed the full daily ration, ensuring uniform nutrient intake. AFB₁ reduced ruminal total volatile fatty acid concentration by approximately 16% (P = 0.011), with lower acetate (P = 0.014) and propionate (P = 0.017), and increased ruminal ammonia nitrogen (P = 0.018) and lipopolysaccharide (P = 0.009). Plasma activities of aspartate aminotransferase, γ-glutamyl transferase, and glutamate dehydrogenase increased approximately 2.2-, 2.9-, and 2.8-fold, respectively (all P < 0.001), while albumin and total protein decreased. AFB₁ increased TNF-α, IL-6, IL-1β, nitric oxide, and protein carbonyls, and decreased IL-10, IFN-γ, glutathione, superoxide dismutase, and catalase. During intravenous glucose tolerance testing, AFB₁-exposed ewes had lower basal glucose, higher peak glucose, greater incremental glucose area under the curve, prolonged glucose half-life, higher basal non-esterified fatty acids, and attenuated NEFA suppression. These findings indicate that chronic high-level AFB₁ exposure disrupts coordinated rumen–liver–immune–metabolic function and reduces glucose–lipid metabolic flexibility in fat-tailed ewes.

## Highlights

• AFB₁ reduced ruminal total volatile fatty acid concentration in ewes.
• AFB₁ increased plasma liver enzyme activities and reduced albumin.
• AFB₁ shifted inflammatory and oxidative stress biomarkers.
• AFB₁ impaired glucose clearance during IVGTT.
• AFB₁ attenuated NEFA suppression after glucose challenge.

## Graphical abstract

**Figure:**
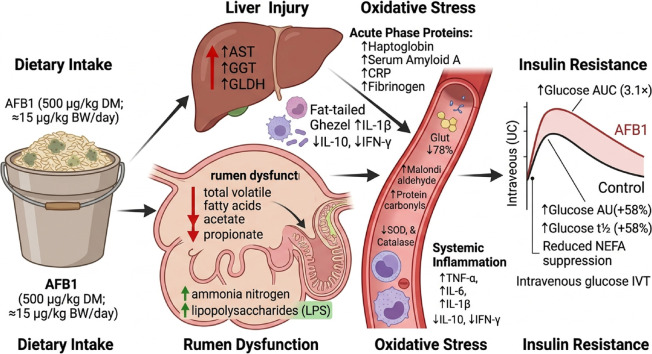

## 1 Introduction

Aflatoxin contamination of livestock feed remains a persistent challenge for animal production, animal health, and food safety. Aflatoxin B₁ (AFB₁), produced mainly by *Aspergillus flavus* and *Aspergillus parasiticus*, is considered the most toxic and biologically important aflatoxin because of its hepatotoxic, immunotoxic, carcinogenic, and productivity-limiting effects (Benkerroum, 2020; Kensler et al., 2011; Marin et al., 2013; Rushing & Selim, 2019). The risk of contamination increases when feed ingredients are produced, transported, or stored under warm and humid conditions, and climate-related changes are expected to increase the geographic risk of AFB₁ contamination in feed crops (Battilani et al., 2016; Eskola et al., 2020; Pinotti et al., 2016).

Ruminants are partly protected from dietary mycotoxins by ruminal microbial transformation, adsorption, and dilution within the rumen ecosystem. However, this protection is incomplete and may be overwhelmed during sustained or high-level exposure (Fink-Gremmels, 2008; Gallo et al., 2015). In ruminants, aflatoxicosis has been associated with impaired feed utilization, reduced performance, altered rumen fermentation, hepatic injury, immune dysfunction, oxidative stress, and carry-over of aflatoxin metabolites into edible products (Gallo et al., 2015; Jiang et al., 2021; Ogunade et al., 2018; Shi et al., 2022; Zhang et al., 2022).

The liver is the primary target organ for AFB₁ toxicity. After absorption, AFB₁ is bioactivated by hepatic cytochrome P450 enzymes to AFB₁-8,9-epoxide, which can bind cellular macromolecules, disrupt mitochondrial function, and initiate oxidative and inflammatory injury (Benkerroum, 2020; Kensler et al., 2011; Rushing & Selim, 2019). In sheep, experimental AFB₁ exposure has been linked with hepatocellular damage, oxidative stress, inflammatory activation, apoptosis, altered gastrointestinal microbial ecology, and impaired tissue function (Cao et al., 2021; Lin et al., 2022; Sui et al., 2022). These responses may affect whole-animal nutrient metabolism because the liver integrates rumen-derived substrates, amino acid catabolism, lipid handling, ureagenesis, glucose production, and acute-phase responses.

Glucose and lipid metabolism are particularly important in ruminants because glucose supply depends largely on hepatic gluconeogenesis rather than direct intestinal glucose absorption. Propionate from ruminal fermentation is a major gluconeogenic precursor, whereas non-esterified fatty acids (NEFA) reflect the balance between adipose tissue lipolysis, insulin-mediated antilipolytic regulation, and hepatic fatty acid handling (Aschenbach et al., 2010; De Koster & Opsomer, 2013). Therefore, ruminal fermentation disruption, hepatic injury, systemic inflammation, and oxidative stress may jointly impair glucose–lipid metabolic flexibility. Pro-inflammatory cytokines such as TNF-α and IL-6 can interfere with insulin-mediated metabolic regulation, while oxidative stress can impair mitochondrial and cellular signaling pathways involved in energy metabolism (Hotamisligil, 2006; Kvidera et al., 2017). However, without dynamic insulin responses or tissue-level insulin-signaling measurements, changes in glucose and NEFA kinetics should be interpreted as altered glucose–lipid metabolic regulation rather than definitive evidence of insulin resistance.

Fat-tailed sheep provide a relevant model for examining these interactions. In these breeds, a large proportion of body lipid is stored in the tail depot, which contributes to energy adaptation under challenging environmental and nutritional conditions (Khachadurian et al., 1966). Because lipid mobilization and glucose metabolism are tightly linked in ruminants, toxicant-induced disruption of hepatic and adipose-related metabolic regulation may be especially important in fat-tailed breeds. Nevertheless, direct responses of tail adipose tissue to AFB₁ exposure have not been fully characterized, and mechanisms involving adipose tissue inflammation, oxidative injury, or insulin signaling remain biologically plausible but require direct tissue-level investigation.

The objective of this study was to evaluate the effects of chronic high-level dietary AFB₁ exposure on rumen fermentation, hepatic function, inflammatory and oxidative stress biomarkers, and glucose and NEFA kinetics during an intravenous glucose tolerance test (IVGTT) in fat-tailed Ghezel ewes. We hypothesized that AFB₁ exposure would disrupt ruminal fermentation and hepatic function, provoke systemic inflammatory–oxidative responses, and impair glucose–lipid metabolic flexibility. The findings are intended to improve understanding of the integrated rumen–liver–immune–metabolic consequences of chronic aflatoxicosis in fat-tailed sheep.

## 2 Materials and methods

### 2.1 Ethics statement and experimental location

The on-farm portion of the study was carried out between April and July 2018 at the Sheep Research Unit of the Educational-Research Farm, Department of Animal Science, Faculty of Agriculture, Urmia University, Iran (37.5287°N, 45.0469°E). The study consisted of a 6-d adaptation period followed by a 40-d experimental period. All procedures were approved by the Animal Care and Use Committee of Urmia University (Approval No. IR3014185) and were conducted in accordance with institutional guidelines for animal care and use.

Laboratory analyses were performed at the Animal Nutrition Laboratory, Department of Animal Science, Urmia University; the Central Laboratory of Urmia University; Nobel Food Industry Laboratory, Tabriz, Iran; and Ayandeh Veterinary Laboratory. Cultivation of *Aspergillus flavus* and production of AFB₁-contaminated rice culture were performed in the Department of Animal Science, Urmia University.

### 2.2 Production and quantification of AFB₁-contaminated rice culture

A lyophilized *Aspergillus flavus* strain was obtained from the Iranian Research Organization for Science and Technology Culture Collection, Tehran, Iran. The isolate was reactivated on potato dextrose agar. Mature sporulating colonies were rinsed with sterile 0.005% Triton X-100, gently scraped, and homogenized to prepare a conidial suspension.

AFB₁ production was carried out by solid-state fermentation on polished rice using a protocol adapted from Shotwell et al. (1966). Briefly, 50 g of long-grain rice and 25 mL of distilled water were placed in 300-mL Erlenmeyer flasks, hydrated for 2 h, sterilized at 121°C for 20 min, cooled to room temperature, inoculated with 0.5 mL of conidial suspension, and incubated at 28°C for 6 d. Sterile distilled water was added during incubation to maintain substrate moisture. After incubation, the mold-colonized rice was homogenized and analyzed for aflatoxin concentration.

Aflatoxins were quantified by high-performance liquid chromatography (HPLC) with post-column derivatization and fluorescence detection using a YL91100 HPLC system (Young Lin Instrument Co., Ltd., Republic of Korea) equipped with an Agilent Zorbax Eclipse XDB-C18 column (150 mm × 4.6 mm, 5 µm; Agilent Technologies, Santa Clara, CA, USA). The mobile phase consisted of water, methanol, and acetonitrile. Quantification followed the Iranian National Standard protocol (ICS:67.040) and included immunoaffinity column clean-up. Certified AFB₁ and AFG₁ reference standards were used for calibration. Several batches of solid-state fermented rice were produced; all inoculated rice was pooled, thoroughly mixed, and three representative aliquots were analysed. The final culture material contained 748.2 ± 15.0 µg total aflatoxins/g (mean ± SD, n = 3), of which 739.7 ± 14.8 µg/g was AFB₁.

### 2.3 Dose rationale

The dietary AFB₁ concentration of 500 µg/kg dry matter (DM) was selected as a high-level chronic challenge expected to induce measurable physiological disruption without producing acute lethality. This dose is approximately 60-fold lower than the acute oral LD₅₀ of ∼1 mg/kg BW reported for sheep (Cao et al., 2021; Sui et al., 2022). Similar dietary concentrations have been used in small-ruminant studies to evaluate AFB₁ effects on nutrient utilization, metabolism, and tissue injury (Gowda et al., 2007; Shi et al., 2022; Zhang et al., 2022). For mature ewes weighing approximately 57 kg and receiving approximately 1.5 to 1.8 kg DM/d, this dietary concentration corresponded to an estimated daily exposure of approximately 13 to 16 µg AFB₁/kg body weight. This dose should be interpreted as an experimental toxicological challenge rather than as a safe or typical field exposure.

### 2.4 Animals, housing, and experimental design

Twenty clinically healthy, non-pregnant, non-lactating multiparous Ghezel ewes were enrolled in a completely randomized design. The ewes averaged 57 ± 2 kg body weight and had body condition scores between 3.0 and 3.5 on a 1-to-5 scale. Each ewe was considered an experimental unit, providing 10 replicates per dietary treatment. This addition makes the replication structure explicit and addresses the reviewer’s request.

Before the experiment, all animals underwent physical examination, body -weight recording, and routine antiparasitic treatment according to farm practice. Non-pregnancy was confirmed by transabdominal ultrasonography.

Ewes were housed individually in metabolic crates measuring 2.0 × 1.5 m with free access to water. During adaptation, all animals were gradually accustomed to the basal ration and housing conditions without AFB₁ exposure. After adaptation, ewes were randomly assigned to one of two dietary treatments: control (CON; n = 10) or AFB₁-exposed (AFB₁; n = 10). The basal total mixed ration (Table 1) was formulated using SRNS V 1.9 software and restricted fed (approximately 1.1 kg/d) to meet approximately 120% of maintenance energy and crude protein requirements for mature ewes according to NRC (2007).

**Table 1:** Ingredient composition of the total mixed ration (DM basis).

Item	g/100 g DM
Alfalfa hay- Full bloom	20
Corn stack silage	20
Wheat straw	24
Wheat bran	20
Corn, ground	10
Soybean meal	5
Calcium carbonate	0.5
Sodium chloride	0.3
Mineral-vitamin premix¹	0.2
**Nutrient composition**	
Dry matter (% as-fed)	68.3
Metabolizable energy (Mcal kg⁻¹ DM)	2.17
Crude protein (% DM)	12.0
Ether extract (% DM)	2.6
Neutral detergent fibre (% DM)	58.9
Non-fibre carbohydrate (% DM)	20.5

The control group received the basal diet without AFB₁, whereas the AFB₁ group received the same basal diet supplemented with AFB₁-contaminated rice culture. The control group received an equivalent amount of uncontaminated rice with the same flavoring used in the AFB₁ dosing mixture. The mass of AFB₁-contaminated rice culture needed to deliver 500 µg AFB₁ per kg of total dietary DM was calculated daily from the known AFB₁ concentration in the pooled rice. The required rice culture was thoroughly mixed with a portion of the daily diet. A few drops of vanilla flavoring were added immediately before offering. The flavored mixture was provided individually before the remaining diet; all animals consumed the entire portion within minutes, and no refusals were observed. Basal diet samples were collected every 10 d and analyzed by HPLC; no detectable AFB₁ was found in the basal ration at the method detection limit of 1 µg/kg DM.

Because the experimental animals were mature, non-pregnant, non-lactating ewes and were not fed ad libitum, no measurable growth was anticipated. The study was designed to evaluate metabolic, oxidative, inflammatory, and endocrine endpoints rather than production performance. However, initial and final body weights of individual ewes were measured on the farm using a calibrated livestock scale with 0.05 kg precision and are reported in Table 2. The total mixed ration was offered at a fixed daily amount and was completely consumed by all ewes with no refusals; individual dry matter intakes were uniform and could not be compared between treatments. Consequently, potential effects of AFB₁ on voluntary feed intake could not be assessed, and rumen fermentation and metabolic responses should be interpreted in the context of a controlled, equal feed supply.

**Table 2-:** Body weight (kg) of fat-tailed ewes at the start and end of the 40-day experimental period.

Variable	Control (n = 10)	AFB₁ (n = 10)	SEM	P-value (Treatment)
Initial BW (day 1)	57.20	57.40	0.800	0.86
Final BW (day 40)	57.00	56.30	0.900	0.58
Δ BW (day 40 – day 1)	–0.20	–1.10	0.600	0.15
*Within-group P-value (paired t-test)*	*0.72*	*0.08*		

### 2.5 Blood sampling and plasma biochemical analyses

On day 30 of the experiment, each ewe was fitted with an indwelling jugular catheter (14 G × 13 cm) to facilitate repeated blood sampling. Jugular blood was collected before the morning feeding on days 31 through 35. Samples were drawn into appropriate evacuated tubes for plasma or serum preparation, immediately placed on ice, and centrifuged at 3,000 × *g* for 15 min at 4°C. Plasma or serum aliquots were stored at −20°C until analysis. The IVGTT was carried out on experimental days 35 and 37.

Plasma metabolites and hormone concentrations were quantified using commercially available diagnostic kits in combination with validated spectrophotometric and enzyme-linked immunosorbent assay procedures. All measurements were run in duplicate, and intra- and inter-assay coefficients of variation were recorded for each analyte.

#### 2.5.1 Energy metabolites and liver function biomarkers

Plasma glucose, cholesterol, blood urea nitrogen (BUN), albumin, total protein, triacylglycerol, creatinine, bilirubin, and activities of alanine aminotransferase (ALT), aspartate aminotransferase (AST), lactate dehydrogenase (LDH), alkaline phosphatase (ALP), γ-glutamyl transferase (GGT), and glutamate dehydrogenase (GLDH) were assessed using an automated clinical chemistry analyzer (BT1500, Biotecnica Instruments, Rome, Italy) and commercial reagent kits (BiorexFars Laboratories, Shiraz, Iran). Globulin concentration was calculated as total protein minus albumin, and the albumin-to-globulin ratio was calculated accordingly.

Non-esterified fatty acids were quantified using an enzymatic colorimetric procedure (BiorexFars Laboratories, Shiraz, Iran). β-Hydroxybutyrate (BHB) was determined using a kinetic enzymatic assay (Randox Laboratories). Glycerol concentration was measured using a commercial glycerol assay kit (Sigma-Aldrich, St. Louis, MO, USA). Plasma insulin was analyzed using an ovine-specific insulin ELISA kit (Mercodia AB, Uppsala, Sweden), with intra- and inter-assay CVs of 3.2% and 5.8%, respectively.

#### 2.5.2 Inflammatory cytokines and acute-phase proteins

Circulating cytokines and acute-phase proteins were determined using commercially available ELISA or immunoturbidimetric assays. Plasma TNF-α was quantified with an ovine-specific ELISA kit (Bioassay Technology Laboratory, Shanghai, China; Cat. No. E0019Bo), with intra- and inter-assay CVs of 1.53% and 4.18%, respectively. Serum amyloid A (SAA) was measured using an ovine ELISA kit from the same manufacturer (Cat. No. E0023Bo), with intra- and inter-assay CVs of 2.18% and 6.32%, respectively.

Plasma IL-6 and IFN-γ were analyzed using ovine-specific ELISA kits supplied by Karmania Pars Gene (Kerman, Iran), with intra- and inter-assay CVs of 1.43% and 3.24% for IL-6 and 2.18% and 2.76% for IFN-γ, respectively. Plasma IL-1β and IL-10 were determined using ovine-specific ELISA kits (MyBioSource, San Diego, CA, USA). Serum haptoglobin concentration was measured using an anti-ovine immunoturbidimetric assay (Biorex Fars, Shiraz, Iran). Ceruloplasmin concentration was determined spectrophotometrically by measuring p-phenylenediamine oxidase activity according to Sunderman and Nomoto (1970). ELISA assays were read using a microplate reader (DANA 3200, Garny, Iran).

#### 2.5.3 Oxidative stress biomarkers

Systemic redox status was assessed using oxidative stress biomarkers and antioxidant enzyme activities. Malondialdehyde (MDA), an indicator of lipid peroxidation, was measured using the thiobarbituric acid reactive substances method (Ohkawa et al., 1979). Reduced glutathione (GSH) was quantified using the 5,5′-dithiobis-2-nitrobenzoic acid reaction (Beutler et al., 1963). Protein carbonyls were determined using the 2,4-dinitrophenylhydrazine derivatization method (Levine et al., 1990). Nitric oxide production was estimated by measuring nitrite and nitrate after enzymatic reduction and Griess reaction (Miranda et al., 2001). Superoxide dismutase (SOD) and catalase (CAT) activities were determined using commercial spectrophotometric kits, with CAT activity assessed according to the principle described by Aebi (1984).

### 2.6 Intravenous glucose tolerance test

To evaluate glucose and lipid metabolic dynamics, an IVGTT was performed on experimental days 35 and 37 following protocols adapted for ovine metabolic studies (Hossein Yazdi et al., 2023; Pires et al., 2007). Feed was withdrawn for 12 h before each test, while water remained available. A sterile 50% dextrose solution was administered as a rapid intravenous bolus at 0.25 g glucose/kg body weight through an indwelling jugular catheter. The glucose bolus was delivered over approximately 30 to 60 s, and the end of infusion was defined as time. The glucose dose of 0.25 g/kg BW was selected based on a validated protocol for fat-tailed sheep (Hossein Yazdi et al., 2023), in which this dose reliably stimulated insulin secretion and revealed physiologically meaningful differences in glucose and NEFA dynamics without causing hyperosmolar phlebitis.

Serial blood samples were collected from the contralateral jugular vein at −5 min and at 5, 10, 15, 20, 25, 30, 40, 45, 60, 75, 90, 120, and 150 min after glucose administration. Samples were immediately placed on ice and centrifuged at 3,000 × *g* for 15 min at 4°C within 30 min of collection. Plasma was aliquoted and stored at −20°C until analysis of glucose and NEFA concentrations.

Basal glucose and NEFA concentrations were determined from the pre-infusion sample. Peak glucose was defined as the highest concentration measured after glucose administration. Incremental glucose area under the curve (AUC) above baseline was calculated using the linear trapezoidal method from 0 to 60 min and from 0 to 120 min and expressed as mg/dL × min. The fractional glucose clearance rate was estimated from the slope of the natural logarithm of glucose concentration during the linear elimination phase, and glucose half-life was calculated as ln(2)/k. NEFA suppression AUC was calculated as the absolute area below baseline from 0 to 60 min and from 0 to 120 min; therefore, larger positive values indicate greater suppression of circulating NEFA after glucose administration.

### 2.7 Rumen fluid collection and analysis

Rumen fluid was collected from each ewe on days 39 and 40 approximately 4 h after the morning meal using a flexible orogastric tube fitted with a perforated brass strainer. Approximately 200 mL of rumen fluid was aspirated using a vacuum pump, with the initial portion discarded to reduce salivary contamination. Ruminal pH was measured immediately using a calibrated portable pH meter. The fluid was strained through cheesecloth, and subsamples were centrifuged and preserved for analysis of volatile fatty acid (VFA) profiles, ammonia nitrogen, and lipopolysaccharide (LPS).

Ammonia nitrogen concentration was quantified using the phenol–hypochlorite colorimetric method (Broderick & Kang, 1980). Individual and total VFA concentrations were analyzed by gas chromatography using a 6820 Gas Chromatograph (Agilent Technologies, Santa Clara, CA, USA) equipped with a flame ionization detector and an HP-FFAP capillary column (30 m × 0.25 mm × 0.25 µm). Helium served as the carrier gas. Peaks were identified using external standards of acetate, propionate, butyrate, iso-butyrate, valerate, and isovalerate (Sigma-Aldrich, St. Louis, MO, USA). Total VFA concentration was calculated as the sum of individual acids, and molar percentages were derived accordingly. Rumen fluid was filtered through four layers of cheesecloth and centrifuged at 11,000 g for 15 min at 4 °C. The supernatant was used directly for lipopolysaccharide (LPS) quantification. LPS concentration was measured with a kinetic turbidimetric Limulus amebocyte lysate (LAL) assay (Bioendo KT Endotoxin Test Kit; Bioendo, Fujian, China), following the procedures described by Hirabayashi et al. (2017). Briefly, a standard curve was prepared with the certified reference endotoxin supplied in the kit, and the assay was performed on a temperature-controlled microplate reader. The rate of turbidity change was monitored, and endotoxin concentrations were calculated from the standard curve. Results are expressed as endotoxin units (EU) per mL of rumen fluid.

### 2.8 Statistical analysis

The individual ewe was considered the experimental unit. Data were analyzed using the MIXED procedure of SAS version 9.4 (SAS Institute Inc., Cary, NC, USA). Incremental glucose AUC during the IVGTT was considered the primary outcome; rumen fermentation variables, plasma metabolites, liver enzyme activities, inflammatory markers, oxidative stress biomarkers, and NEFA responses were considered secondary outcomes. The required sample size was determined a priori using the POWER procedure of SAS (Version 9.4). Calculations were based on the primary outcome variable, the incremental area under the glucose curve (AUC) during the intravenous glucose tolerance test (IVGTT). According to previous reports (Hossein Yazdi et al., 2023; Zhang et al., 2022), dietary AFB₁ exposure was expected to increase AUC by approximately 30%, from 4,800 ± 750 to 6,240 ± 750 (mg/dL) × min, corresponding to Cohen’s d = 1.92. Power analysis for a two‑sample t test assuming equal variances, α = 0.05, and 80% statistical power indicated a minimum requirement of six animals per treatment group (numerator df = 1, denominator df = 10; achieved power = 0.81). To minimize potential losses and improve the precision of secondary outcomes, ten ewes were assigned to each treatment (Control, n = 10; Aflatoxin, n = 10), which increased the achieved power to >0.98 (denominator df = 18).

Before analysis, residuals were evaluated for normality using the Shapiro–Wilk test and Q–Q plots, and homogeneity of variance was assessed using Levene’s test. When required, data were logarithmically or square-root transformed before analysis. Least squares means are presented in the original measurement units, whereas P-values are based on transformed data when transformation was applied. Outliers were screened using studentized residuals and diagnostic plots; no observations were excluded unless a clear analytical or recording error was identified. Variables measured once or summarized as one value per ewe, including rumen fermentation variables and derived IVGTT kinetic variables, were analyzed with treatment as the fixed effect (Yᵢⱼ = μ + Tᵢ + Aⱼ₍ᵢ₎ + εᵢⱼ.; Tᵢ represents treatment and Aₖ₍ᵢ₎ represents the random effect of animal within treatment). For blood variables collected across sampling days and for glucose and NEFA concentrations measured across IVGTT sampling minutes, repeated-measures mixed models were used with treatment, time, and treatment × time as fixed effects and ewe as the repeated subject (Yᵢⱼₖ = μ + Tᵢ + β(Wₖ – W̄) + Dⱼ + (T × D)ᵢⱼ + Aₖ₍ᵢ₎ + εᵢⱼₖ; Tᵢ represents treatment, Dⱼ denotes time (day or minute), β(Wₖ – W̄) accounts for the centered covariate initial body weight was included as a covariate when it improved model fit., and Aₖ₍ᵢ₎ represents the random effect of animal within treatment). As IVGTT was performed on two day, test day was included in the model, or values were averaged within ewe before analysis when no day effect or treatment × day interaction was detected. Covariance structures, including compound symmetry, first-order autoregressive, heterogeneous first-order autoregressive, and unstructured covariance, were compared, and the final structure was selected according to the lowest corrected Akaike information criterion. Based on AICC, the AR(1) covariance structure was selected for all daily longitudinal outcomes, while the ARH(1) structure was chosen for the unequally spaced IVGTT data to accommodate time-dependent variance heterogeneity. Denominator degrees of freedom were estimated using the Kenward–Roger method. Initial body weight was tested as a covariate and retained only when it improved model fit and did not alter the biological interpretation of treatment effects. When treatment × time interactions were significant, treatment effects were evaluated within time point using sliced least squares means. Tukey–Kramer adjustment was applied to pairwise comparisons of least-squares means when the corresponding overall F-test was significant (P ≤ 0.05). This adjustment was used for all repeated-measures outcomes (plasma metabolites, cytokines, liver enzymes, oxidative stress markers, IVGTT variables) and for rumen VFA and LPS concentrations where multiple time-point or treatment comparisons were made. Significance was declared at P ≤ 0.05, and tendencies were discussed at 0.05 < P ≤ 0.10. Because several biomarker panels were evaluated, secondary outcomes were interpreted according to effect size, directionality, and biological consistency rather than isolated P-values alone.

## 3 Results

Initial and final body weights did not differ significantly between treatments (Table 2). Within-group comparisons showed that weight remained stable in the Control group (P = 0.72), while a slight numerical decline was observed in the AFB₁ group (–1.10 kg; P = 0.08).

### 3.1 Rumen fermentation

Dietary AFB₁ exposure altered ruminal fermentation characteristics (Table 3). Ruminal pH did not differ between treatments (P = 0.63). Ammonia nitrogen concentration was greater in AFB₁-exposed ewes than in controls (P = 0.02). Total VFA concentration decreased by approximately 16% in the AFB₁ group (P = 0.01). This reduction was associated with lower acetate (P = 0.01) and propionate (P = 0.01) concentrations, whereas butyrate concentration was not affected (P = 0.581). Isobutyrate and isovalerate concentrations were greater in AFB₁-exposed ewes (P = 0.022 and P = 0.018, respectively). The acetate-to-propionate ratio increased modestly in the AFB₁ group (P = 0.044), and ruminal LPS concentration was higher in AFB₁-exposed ewes than in controls (P = 0.009).

**Table 3:** Effects of dietary aflatoxin B₁ (AFB₁) contamination on ruminal fermentation parameters, volatile fatty acid (VFA) profile, and ruminal lipopolysaccharide (LPS) concentration in fat-tailed ewes.

Parameter	Control	AFB₁	SEM	P-value
Ruminal pH	6.42	6.38	0.06	0.632
Ammonia nitrogen (NH₃-N), mg/dL	14.2	18.7	1.24	0.018
Total VFA, mM	98.5	82.3	4.12	0.011
**Individual VFA (m*M*)**				
Acetate	61.2	50.4	2.85	0.014
Propionate	21.8	16.9	1.32	0.017
Butyrate	11.4	10.8	0.78	0.581
Isobutyrate	1.62	2.14	0.15	0.022
Isovalerate	1.48	1.96	0.13	0.018
Valerate	1.01	1.08	0.09	0.587
**Molar Proportions (mol/100 mol)**				
Acetate	62.1	61.2	0.82	0.041
Propionate	22.1	20.5	0.65	0.038
Butyrate	11.6	13.1	0.48	0.029
BCVFA¹	4.22	5.20	0.22	0.005
Acetate: Propionate Ratio	2.81	2.98	0.09	0.044
Ruminal LPS, EU/mL × 10³	18.4	28.7	2.65	0.009

^1^BCVFA = branched-chain volatile fatty acids (sum of iso-butyrate and iso-valerate).

Data are presented as least squares means. SEM = standard error of the mean. P-values ≤ 0.05 are considered statistically significant.

### 3.2 Plasma metabolites and hepatic function biomarkers

AFB₁ exposure altered circulating metabolites and hepatic function indicators (Table 4, Table 5). Plasma glucose concentration was lower in AFB₁-exposed ewes than in controls (P < 0.001), whereas NEFA concentration was higher (P = 0.009). Plasma BHB and basal insulin concentrations did not differ between treatments (P = 0.567 and P = 0.230, respectively). BUN increased in response to AFB₁ exposure (P < 0.001), and glycerol tended to increase (P = 0.070). Triacylglycerol concentration was not affected (P = 0.327), whereas cholesterol concentration increased modestly in the AFB₁ group (P = 0.024).

**Table 4:** Effect of dietary aflatoxin B1 (AFB1) on plasma metabolite concentrations in fat-tailed ewes.

Parameter	Control (n = 10)	AFB₁ (n = 10)	SEM	P-value
Glucose (mg/dL)	74.47	68.37	0.623	<0.001
NEFA (mmol/L)	0.387	0.477	0.0023	0.001
BHB (mmol/L)	0.325	0.338	0.0293	0.57
Insulin (ng/mL)	11.67	11.81	0.107	0.23
Blood Urea Nitrogen (mmol/L)	4.19	4.85	0.039	<0.001
Glycerol (mg/dL)	0.163	0.197	0.0114	0.07
Triacylglycerol (mg/dL)	5.27	5.20	0.045	0.33
Cholesterol (mg/dL)	59.49	61.98	0.525	0.02
Total protein (g/dL)	5.939	5.083	0.0479	<0.001
Albumin (g/dL)	3.889	2.556	0.0216	<0.001
Globulin (g/dL)	2.051	2.528	0.0603	0.01
Albumin: globulin ratio	1.896	1.011	0.1022	<0.001
Bilirubin (mg/dL)	0.1730	0.1768	0.015	0.22
Creatinine (mg/dL)	0.6915	0.7049	0.0061	0.13

NEFA = non-esterified fatty acids; BHB = β-hydroxybutyrate; SEM = standard error of the mean.

**Table 5:** Effect of dietary aflatoxin B_1_ (AFB1) on plasma liver enzyme activities in fat-tailed ewes.

Parameter	Control (n = 10)	AFB₁ (n = 10)	SEM	P-value
Alanine Aminotransferase (ALT; U/L)	10.29	13.48	0.077	<0.001
Aspartate Aminotransferase (AST; U/L)	77.13	167.18	0.554	<0.001
Lactate Dehydrogenase LDH (U/L)	305.27	445.84	2.911	<0.001
Alkaline Phosphatase ALP(U/L)	97.03	129.80	0.504	<0.001
Gamma-Glutamyl Transferase (GGT; U/L)	33.47	97.21	0.241	<0.001
Glutamate Dehydrogenase (GLDH; U/L)	13.16	37.24	0.131	<0.001

Total protein and albumin concentrations were lower in AFB₁-exposed ewes (P < 0.001 for both), while globulin concentration increased (P = 0.013). Consequently, the albumin-to-globulin ratio was reduced in the AFB₁ group (P < 0.001). Bilirubin and creatinine were not affected by treatment (P = 0.218 and P = 0.138, respectively). AFB₁ exposure increased plasma activities of ALT, AST, LDH, ALP, GGT, and GLDH (all P < 0.001). The most pronounced relative increases were observed for AST, GGT, and GLDH, which increased approximately 2.2-, 2.9-, and 2.8-fold, respectively.

### 3.3 Inflammatory and oxidative stress biomarkers

AFB₁ exposure shifted circulating inflammatory status toward a pro-inflammatory profile (Table 6). Concentrations of TNF-α, IL-6, and IL-1β were higher in AFB₁-exposed ewes than in controls (P < 0.001), whereas IL-10 and IFN-γ concentrations were lower (P = 0.009 and P = 0.003, respectively). Haptoglobin and ceruloplasmin concentrations were higher in AFB₁-exposed ewes than in controls (P = 0.022 and P = 0.005, respectively), whereas SAA concentration did not differ between treatments (P = 0.327).

**Table 6:** Effect of dietary aflatoxin B1 (AFB_1_) on plasma cytokine concentrations and acute phase proteins in fat-tailed ewes.

Parameter	Control	AFB₁	SEM	P-value
**Cytokines**				
TNF-α (pg/mL)	86.87	154.65	3.02	<0.001
IL-6 (pg/mL)	34.88	218.96	5. 22	<0.001
IL-1β (pg/mL)	26.28	148.24	9.82	<0.001
IL-10 (pg/mL)	13.15	5.21	2.213	0.009
IFN-γ (pg/mL)	84.35	31.26	12.16	0.003
**Acute Phase Proteins**				
Haptoglobin (mg/dL)	13.09	15.62	0.43	0.023
Serum Amyloid A (SAA; mg/L)	18.24	19.32	4.15	0.331
Ceruloplasmin (mg/dL)	18.34	38.26	4.632	0.005

AFB₁ exposure also altered oxidative stress biomarkers (Table 7). Nitric oxide (NO), MDA, and protein carbonyl concentrations were higher in AFB₁-exposed ewes than in controls (P < 0.001, P = 0.022, and P < 0.001, respectively). In contrast, GSH concentration and SOD and CAT activities were lower in the AFB₁ group (all P < 0.001). These changes indicate increased oxidative and nitrosative stress with reduced antioxidant defense capacity.

**Table 7:** Effect of dietary aflatoxin B_1_ (AFB1) on plasma oxidative stress indices in fat-tailed ewes.

Parameter	Control (n = 10)	AFB₁ (n = 10)	SEM	P-value
Nitric oxide (NO; nmol/mg protein)	6.14	10.59	0.20	<0.001
Malondialdehyde (MDA; nmol/mL)	1.86	3.18	0.15	0.022
Glutathione (GSH; μmol/L)	8.831	1.938	0.91	<0.001
Protein carbonyl Content (nmol/mL)	7.31	13.27	1.02	<0.001
Superoxide dismutase (SOD; U/mL)	5.94	5.08	0.05	<0.001
Catalase (CAT; U/mL)	26.54	14.57	0.60	<0.001

### 3.4 Glucose and NEFA kinetics during IVGTT

**Fig. 1:**
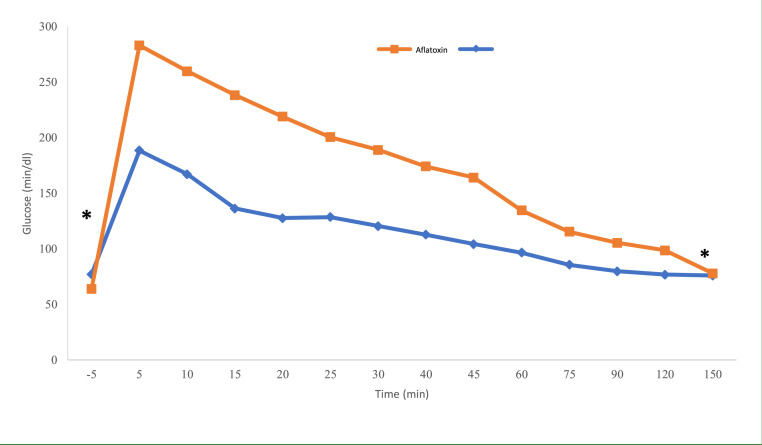
Plasma glucose concentrations during the intravenous glucose tolerance test in control and AFB₁-exposed fat-tailed ewes. Values are presented as least squares means. AFB₁-exposed ewes showed higher peak glucose concentration and slower glucose clearance after intravenous glucose administration. Asterisks (*) denote non-significant differences between AFB1 and control groups at the corresponding time point (-5 and 150 minutes; P > 0.05). all other time points show significant differences (P < 0.05).

AFB₁ exposure altered glucose kinetics during IVGTT (Table 8 and Fig. 1). Basal glucose concentration was lower in AFB₁-exposed ewes (P < 0.001), but peak glucose concentration after intravenous glucose administration was higher (P < 0.001). Incremental glucose AUC from 0 to 60 min and from 0 to 120 min was greater in the AFB₁ group (P < 0.001), and glucose clearance rate was lower (P < 0.001). Glucose half-life was also prolonged in AFB₁-exposed ewes (P < 0.001). These responses indicate impaired glucose clearance after the glucose challenge.

**Table 8:** Effect of dietary aflatoxin B1 (AFB1) on glucose kinetics during intravenous glucose tolerance test (IVGTT) in fat-tailed ewes.

Parameter	Control (n = 10)	AFB₁ (n = 10)	SEM	P-value
Basal Glucose (G₀; mg/dL)	76.87	62.42	1.63	<0.001
Peak Glucose (Gₚₑₐₖ; mg/dL)	188.39	293.49	10.9	<0.001
AUC Glucose 0–60 min (AUCG₆₀; mg/dL × 60 min)	1215.65	3472.89	71.7	<0.001
AUC Glucose 0–120 min (AUCG₁₂₀; mg/dL × 120 min)	1244.19	3826.70	80.4	<0.001
Glucose clearance rate_60 min_(%/min)	3.187	2.008	0.15	<0.001
Glucose Half-Life (T₁/₂; min)	22.25	35.27	1.67	<0.001

AUC = area under the curve.

**Fig. 2:**
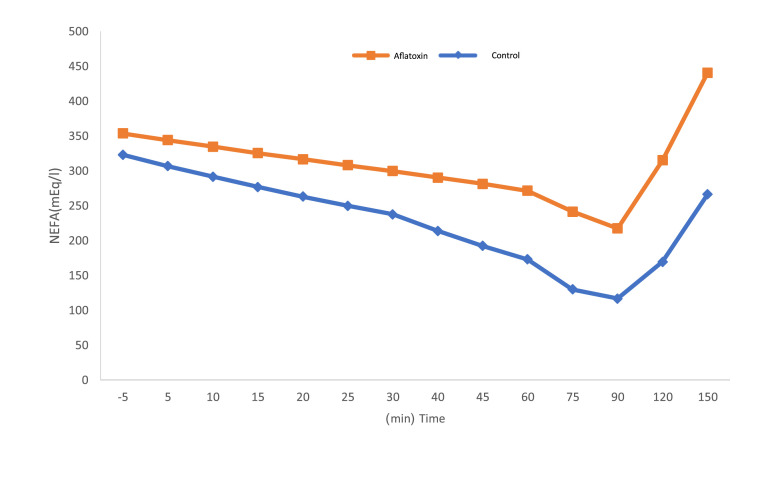
Plasma non-esterified fatty acid concentrations during the intravenous glucose tolerance test in control and AFB₁-exposed fat-tailed ewes. Values are presented as least squares means. AFB₁-exposed ewes had higher basal NEFA concentration and attenuated NEFA suppression after glucose administration. All of the differences between AFB1 and control groups at the corresponding time points were statistically significant (P < 0.05).

AFB₁ exposure also modified NEFA dynamics during IVGTT (Table 9 and Fig. 2). Basal NEFA concentration was higher in AFB₁-exposed ewes (P < 0.001). After glucose administration, NEFA suppression was attenuated in the AFB₁ group, as indicated by lower NEFA suppression AUC from 0 to 60 min and from 0 to 120 min (P < 0.001). NEFA clearance rate was also lower in AFB₁-exposed ewes (P < 0.001), indicating a blunted antilipolytic response after glucose challenge.

**Table 9:** Effect of dietary aflatoxin B_1_ (AFB1) on non-esterified fatty acid (NEFA) kinetics following intravenous glucose tolerance test (IVGTT) in fat-tailed ewes.

Parameter	Control (n = 10)	AFB₁ (n = 10)	SEM	P-value
Basal NEFA (NEFA₀; µEq/L)	322.75	357.57	3.331	<0.001
AUC NEFA 0–60 min (AUCNEFA₆₀; µEq/L × 60 min)	-1754.04	-938.28	29.731	<0.001
AUC NEFA 0–120 min (AUCNEFA₁₂₀; µEq/L × 120 min)	-3135.31	-1573.70	44.710	<0.001
NEFA Clearance Rate (CR; %/min)	4.185	3.722	0.037	<0.001

AUC = area under the curve.

## 4 Discussion

This study demonstrates that chronic high-level dietary AFB₁ exposure disrupts multiple interconnected physiological systems in fat-tailed ewes. AFB₁ exposure altered rumen fermentation, induced marked hepatic injury, promoted systemic inflammation and oxidative stress, and impaired glucose and NEFA dynamics during IVGTT. Together, these findings support the conclusion that AFB₁ compromises coordinated rumen–liver–immune–metabolic function in sheep.

### 4.1 Rumen fermentation disruption during AFB₁ exposure

AFB₁ exposure reduced total ruminal VFA concentration and decreased acetate and propionate concentrations without changing ruminal pH. This pattern indicates impaired fermentation output rather than acidotic fermentation. Previous work in ruminants has shown that AFB₁ can alter rumen fermentation, nutrient digestibility, and microbial activity (Gallo et al., 2015; Ogunade et al., 2018; Shi et al., 2022; Zhao et al., 2022). In the present study, lower total VFA suggests reduced fermentative efficiency, while lower propionate is especially relevant because propionate is a major gluconeogenic precursor in ruminants (Aschenbach et al., 2010).

The increase in ammonia nitrogen and branched-chain VFA suggests altered nitrogen metabolism and amino acid fermentation. This may reflect impaired microbial nitrogen capture, altered proteolysis, or reduced synchrony between carbohydrate and nitrogen fermentation. Ruminal LPS concentration was also higher in AFB₁-exposed ewes. Because plasma LPS, ruminal epithelial integrity, and rumen microbial composition were not measured, this finding should be interpreted cautiously as evidence of ruminal disturbance rather than proof of systemic endotoxin translocation or ruminal epithelial barrier failure. These mechanisms are biologically plausible but were not directly measured in the present study.

### 4.2 Hepatic injury as a central component of the systemic response

The liver is the major site of AFB₁ bioactivation and detoxification. In the present study, AFB₁ exposure increased ALT, AST, LDH, ALP, GGT, and GLDH activities, indicating hepatocellular and hepatobiliary injury. The marked increases in AST and GGT are consistent with hepatocellular damage and cholestatic or biliary involvement, whereas increased GLDH suggests mitochondrial involvement in hepatic injury. These findings agree with previous reports showing that AFB₁ exposure damages the liver through oxidative stress, inflammation, apoptosis, and mitochondrial dysfunction (Benkerroum, 2020; Kensler et al., 2011; Moloi et al., 2024; Rushing & Selim, 2019; Sui et al., 2022).

The substantial elevations in plasma activities of ALT, AST, LDH, ALP, GGT, and GLDH provide clear evidence of hepatocellular and hepatobiliary injury, mirroring patterns previously documented in sheep exposed to AFB₁ (Cao et al., 2021; Lin et al., 2022; Sui et al., 2022; Zhang et al., 2022). The approximately 2.2-fold increase in AST and 2.9-fold increase in GGT are consistent with the magnitude of transaminase and cholestatic enzyme elevations reported by Sui et al. (2022) in acutely intoxicated sheep, although our chronic model produced these changes without clinical signs. The nearly threefold rise in GLDH is in line with values observed by Cao et al. (2021) and indicates mitochondrial damage within hepatocytes. The mild but significant elevation in bilirubin (P = 0.022; Table 2) further supports impaired hepatic excretory function, a feature also noted in aflatoxin-challenged ruminants (Gallo et al., 2015). These enzyme patterns are consistent with the well-characterized bioactivation of AFB₁ to the reactive AFB₁-8,9-epoxide, which forms adducts with cellular macromolecules and triggers oxidative stress-mediated apoptotic and necrotic injury (Benkerroum, 2020; Kensler et al., 2011; Rushing & Selim, 2019).

The reduction in total plasma protein and albumin, together with the increase in globulin and the consequent decline in the albumin-to-globulin ratio, indicate both impaired hepatic synthetic function and an acute-phase response. Similar reductions in albumin and increases in globulin have been reported in sheep and cattle during aflatoxicosis (Cao et al., 2021; Zhang et al., 2022). The elevation in blood urea nitrogen, accompanied by higher ruminal ammonia nitrogen, points to increased amino acid catabolism and urea synthesis, a pattern previously described in aflatoxin-exposed ruminants (Zhang et al., 2022). In contrast, the stability of plasma creatinine suggests that renal function was largely unaffected, localizing the primary insult to the liver and rumen. The modest increase in plasma cholesterol is consistent with intrahepatic cholestasis, a recognized component of aflatoxin-induced liver injury (Gallo et al., 2015).

Overall, these hepatic and protein-metabolic alterations are in strong agreement with the existing literature and confirm that our chronic exposure model recapitulates the characteristic biochemical fingerprint of aflatoxicosis in small ruminants, while extending the phenotype to include early signs of insulin resistance, as discussed below.

Hepatic injury provides a plausible link between AFB₁ exposure and altered glucose–lipid metabolism. The ruminant liver is central to gluconeogenesis, ureagenesis, fatty acid handling, ketogenesis, and acute-phase protein synthesis. Therefore, AFB₁-induced hepatic injury may contribute to reduced basal glucose availability, impaired glucose clearance, altered lipid handling, and modified inflammatory and oxidative responses.

### 4.3 Systemic inflammation and oxidative stress

AFB₁ exposure increased TNF-α, IL-6, IL-1β, haptoglobin, and ceruloplasmin and decreased IL-10 and IFN-γ. This pattern indicates a systemic inflammatory response characterized by enhanced pro-inflammatory signaling and reduced anti-inflammatory or immunoregulatory tone. Similar inflammatory activation has been reported in sheep and other species exposed to AFB₁ (Cao et al., 2021; Sui et al., 2022). IL-6 and IL-1β are key mediators of the acute-phase response and may have contributed to the increased acute-phase protein response observed here (Ceciliani et al., 2012). In contrast, SAA was not significantly affected, suggesting that acute-phase proteins did not respond uniformly to the AFB₁ challenge.

The oxidative stress profile was also altered. Increased MDA and protein carbonyls indicate lipid and protein oxidation, while increased nitric oxide suggests enhanced nitrosative stress. The marked reduction in GSH and lower SOD and CAT activities indicate depletion or suppression of antioxidant defenses. These findings are consistent with the known mechanism of AFB₁ bioactivation, in which reactive metabolites and mitochondrial dysfunction promote reactive oxygen species formation (Benkerroum, 2020; Kensler et al., 2011; Moloi et al., 2024). Oxidative stress and inflammation likely reinforced each other, contributing to the systemic nature of the response.

Inflammatory and oxidative pathways may contribute to altered glucose and lipid metabolism. Pro-inflammatory cytokines can interfere with insulin-mediated metabolic regulation, stimulate lipolysis, and increase immune glucose demand (Hotamisligil, 2006; Kvidera et al., 2017). Oxidative stress can impair mitochondrial energy metabolism and cellular signaling. However, the present study did not measure tissue insulin-signaling proteins, hepatic gene expression, plasma LPS, ruminal epithelial integrity, or adipose tissue inflammatory markers; therefore, these mechanisms are biologically plausible but were not directly measured in the present study.

### 4.4 Glucose–lipid metabolic disruption during IVGTT

The IVGTT results provide functional evidence that AFB₁ exposure impaired glucose and lipid metabolic flexibility. AFB₁-exposed ewes had lower basal glucose but higher peak glucose, greater incremental glucose AUC, reduced glucose clearance rate, and prolonged glucose half-life after intravenous glucose administration. This pattern indicates impaired glucose clearance after a defined glucose load. The magnitude of the glucose AUC increase (3.1-fold) is comparable to that reported in fat-tailed lambs experiencing negative energy balance (Hossein Yazdi et al., 2023) and in endotoxin-challenged dairy cows (Kvidera et al., 2017), where impaired glucose tolerance was linked to inflammation-driven insulin resistance. The lower basal glucose concentration may be explained partly by reduced ruminal propionate availability and impaired hepatic gluconeogenic capacity due to liver injury. Conversely, slower glucose clearance after the bolus may reflect impaired peripheral glucose disposal, altered hepatic glucose handling, or both. Similar dissociation between basal hypoglycaemia and post-challenge hyperglycaemia has been observed in periparturient dairy cows with subclinical liver dysfunction (De Koster & Opsomer, 2013).

Basal insulin concentration did not differ between treatments. However, basal insulin alone does not allow direct assessment of insulin secretion or insulin sensitivity during glucose challenge. Dynamic insulin measurements during IVGTT would be required to distinguish impaired insulin secretion from impaired insulin action. Nonetheless, previous work in fat-tailed sheep has shown that a 0.25 g/kg glucose dose elicits clear insulin secretory responses and that glucose and NEFA kinetics alone are reliable surrogate indicators of insulin sensitivity (Hossein Yazdi et al., 2023; Pires et al., 2007). Therefore, the present data should be interpreted as evidence of impaired glucose tolerance and altered metabolic regulation rather than definitive proof of insulin resistance.

AFB₁-exposed ewes also had higher basal NEFA concentrations and attenuated NEFA suppression after glucose administration. Because insulin normally suppresses adipose tissue lipolysis after glucose-stimulated insulin release, reduced NEFA suppression suggests impaired antilipolytic regulation. This response is particularly relevant in fat-tailed ewes, where lipid depots contribute substantially to whole-body energy balance. Impaired NEFA suppression has similarly been reported in dairy cows with experimentally induced endotoxemia (Kvidera et al., 2017) and in early-lactation cows exhibiting physiological insulin resistance (De Koster & Opsomer, 2013). The absence of increased BHB despite higher NEFA may indicate that hepatic fatty acid oxidation or ketogenesis did not increase proportionally, potentially because of hepatic mitochondrial impairment. This uncoupling of NEFA supply and ketogenesis has been noted in other hepatotoxic models and in aflatoxin-exposed sheep (Zhang et al., 2022). However, direct hepatic lipid metabolism and adipose tissue responses were not measured.

### 4.5 Limitations

Several limitations should be considered. First, only one AFB₁ dose was tested, preventing dose-response interpretation. Second, although the fixed ration was fully consumed and daily dry matter intake was therefore uniform across animals, individual feed intake was not directly measured. Pair-feeding designs or ad libitum intake monitoring in future studies would help distinguish direct metabolic effects of AFB₁ from those mediated by altered voluntary intake. Third, insulin was measured basally but not dynamically during IVGTT, so insulin secretion and insulin sensitivity could not be directly quantified. Fourth, tissue-level insulin signaling, hepatic histopathology, rumen microbial composition, plasma LPS, ruminal epithelial integrity, and tail adipose tissue responses were not measured. These mechanisms are biologically plausible but were not directly measured in the present study. Finally, the glucose bolus (0.25 g/kg BW) used in the IVGTT was lower than the 0.5–1.0 g/kg dose employed in some ovine studies, which may limit direct quantitative comparisons with those reports. Future work should include these endpoints to improve mechanistic interpretation.

## 5 Conclusions

Chronic high-level dietary AFB₁ exposure was associated with changes in rumen fermentation, hepatic function, inflammatory–oxidative balance, and glucose–lipid metabolism in fat-tailed Ghezel ewes. The AFB₁ challenge reduced total ruminal VFA and propionate, increased ruminal ammonia nitrogen and LPS, induced hepatocellular and hepatobiliary injury, activated systemic inflammation, depleted antioxidant defenses, impaired glucose clearance, and attenuated NEFA suppression during IVGTT. These findings indicate that AFB₁ compromises coordinated rumen–liver–immune–metabolic regulation and reduces glucose–lipid metabolic flexibility in fat-tailed ewes. Because dynamic insulin responses and tissue insulin-signaling endpoints were not measured, the findings should be interpreted as impaired glucose tolerance and attenuated antilipolytic response rather than definitive proof of insulin resistance. Future studies should include multiple AFB₁ doses, tissue-level insulin-signaling endpoints, hepatic histopathology, and rumen microbial profiling to clarify the mechanisms linking aflatoxicosis with systemic metabolic dysfunction.

## Ethics approval

All animal procedures were approved by the Animal Care and Use Committee of Urmia University (Approval No. IR3014185) and were conducted in accordance with institutional animal-care guidelines.

## Declaration of generative AI and AI-assisted technologies in the manuscript preparation process

During the preparation of this work, the authors used AI-assisted language editing to improve clarity, structure, and readability. After using this tool, the authors reviewed and edited the content as needed and take full responsibility for the content of the manuscript.

## Funding

This work was supported by the Urmia University MSc programme [Grant No. 4185]. Kimiya Danesh Alvand Co. provided partial financial support and contributed to laboratory analysis costs. The funders had no role in data interpretation, manuscript preparation, or the decision to submit the manuscript for publication.

## CRediT authorship contribution statement

**Nasibeh Ider:** Software, Investigation, Formal analysis. **Hamed Khalilvandi-Behroozyar:** Writing – review & editing, Supervision, Resources, Project administration, Methodology, Funding acquisition, Conceptualization. **Abdollah Ahmadpour:** Supervision, Formal analysis. **Farhad Farhangpajouh:** Supervision, Formal analysis, Data curation. **Maryam Sahebi-Ala:** Writing – review & editing, Software, Formal analysis. **Yanting Chen:** Writing – review & editing. **Morteza Hosseini Ghaffari:** Writing – review & editing, Formal analysis, Data curation.

## Declaration of competing interest

The authors declare that they have no known competing financial interests or personal relationships that could have appeared to influence the work reported in this paper. Kimiya Danesh Alvand Co. has no commercial interests in toxin binders, mycotoxin testing, or related products.

## Data Availability

The data supporting the findings of this study are available from the corresponding author upon reasonable request.

## References

[bib0001] Aebi H. (1984). Catalase in vitro. Methods in Enzymology.

[bib0002] Aschenbach J.R., Kristensen N.B., Donkin S.S., Hammon H.M., Penner G.B. (2010). Gluconeogenesis in dairy cows: The secret of making sweet milk from sour dough. IUBMB Life.

[bib0003] Battilani P., Toscano P., Van der Fels-Klerx H.J., Moretti A., Camardo Leggieri M., Brera C., Rortais A., Goumperis T., Robinson T. (2016). Aflatoxin B1 contamination in maize in Europe increases due to climate change. Scientific Reports.

[bib0004] Benkerroum N. (2020). Chronic and acute toxicities of aflatoxins: Mechanisms of action. International Journal of Environmental Research and Public Health.

[bib0005] Beutler E., Duron O., Kelly B.M. (1963). Improved method for the determination of blood glutathione. Journal of Laboratory and Clinical Medicine.

[bib0006] Broderick G.A., Kang J.H. (1980). Automated simultaneous determination of ammonia and total amino acids in ruminal fluid and in vitro media. Journal of Dairy Science.

[bib0007] Cao Q.Q., Lin L.X., Xu T.T., Lu Y., Zhang C.D., Yue K., Huang S.C., Dong H.J., Jian F.C. (2021). Aflatoxin B1 alters meat quality associated with oxidative stress, inflammation, and gut-microbiota in sheep. Ecotoxicology and Environmental Safety.

[bib0008] Ceciliani F., Ceron J.J., Eckersall P.D., Sauerwein H. (2012). Acute phase proteins in ruminants. Journal of Proteomics.

[bib0009] De Koster J.D., Opsomer G. (2013). Insulin resistance in dairy cows. Veterinary Clinics of North America: Food Animal Practice.

[bib0010] Eskola M., Kos G., Elliott C.T., Hajšlová J., Mayar S., Krska R. (2020). Worldwide contamination of food-crops with mycotoxins: Validity of the widely cited FAO estimate of 25%. Critical Reviews in Food Science and Nutrition.

[bib0011] Fink-Gremmels J. (2008). Mycotoxins in cattle feeds and carry-over to dairy milk: A review. Food Additives & Contaminants: Part A.

[bib0012] Gallo A., Giuberti G., Frisvad J.C., Bertuzzi T., Nielsen K.F. (2015). Review on mycotoxin issues in ruminants: Occurrence in forages, effects of mycotoxin ingestion on health status and animal performance and practical strategies to counteract their negative effects. Toxins.

[bib0013] Gowda N.K.S., Suganthi R.U., Malathi V., Raghavendra A. (2007). Effect of feeding graded levels of aflatoxin B1 on feed intake, growth and nutrient utilization in sheep. Indian Journal of Animal Sciences.

[bib0014] Hirabayashi H., Kawashima K., Okimura T., Tateno A., Suzuki A., Asakuma S. (2017). Effect of nutrient levels during the far-off period on postpartum productivity in dairy cows. Animal Science Journal.

[bib0015] Hossein Yazdi M., Ganjkhanlou M., Zali A., Sadeghi M. (2023). Fat-tailed and thin-tailed lambs’ responses to glucose and insulin challenges during different energy balances. Journal of Livestock Science and Technologies.

[bib0016] Hotamisligil G.S. (2006). Inflammation and metabolic disorders. Nature.

[bib0017] Jiang Y., Ogunade I.M., Vyas D., Adesogan A.T. (2021). Aflatoxin in dairy cows: Toxicity, occurrence in feedstuffs and milk and dietary mitigation strategies. Toxins.

[bib0018] Kensler T.W., Roebuck B.D., Wogan G.N., Groopman J.D. (2011). Aflatoxin: A 50-year odyssey of mechanistic and translational toxicology. Toxicological Sciences.

[bib0019] Khachadurian A.K., Adrouni B., Yacoubian H. (1966). Metabolism of adipose tissue in the fat tail of the sheep in vivo. Journal of Lipid Research.

[bib0020] Kvidera S.K., Horst E.A., Abuajamieh M., Mayorga E.J., Fernandez M.V., Baumgard L.H. (2017). Glucose requirements of an activated immune system in lactating Holstein cows. Journal of Dairy Science.

[bib0021] Levine R.L., Garland D., Oliver C.N., Amici A., Climent I., Lenz A.G., Ahn B.W., Shaltiel S., Stadtman E.R. (1990). Determination of carbonyl content in oxidatively modified proteins. Methods in Enzymology.

[bib0022] Lin L.X., Cao Q.Q., Zhang C.D., Xu T.T., Yue K., Li Q., Liu F., Wang X.D., Dong H.J., Huang S.C. (2022). Aflatoxin B1 causes oxidative stress and apoptosis in sheep testes associated with disrupting rumen microbiota. Ecotoxicology and Environmental Safety.

[bib0023] Marin S., Ramos A.J., Cano-Sancho G., Sanchis V. (2013). Mycotoxins: Occurrence, toxicology, and exposure assessment. Food and Chemical Toxicology.

[bib0024] Miranda K.M., Espey M.G., Wink D.A. (2001). A rapid, simple spectrophotometric method for simultaneous detection of nitrate and nitrite. Nitric Oxide.

[bib0025] Moloi T.P., Ziqubu K., Mazibuko-Mbeje S.E., Mabasa L., Ndwandwe D., Shabalala S.C., Nkambule B.B., Dludla P.V., Mthembu S.X.H. (2024). Aflatoxin B1-induced hepatotoxicity through mitochondrial dysfunction, oxidative stress, and inflammation as central pathological mechanisms: A review of experimental evidence. Toxicology.

[bib0026] NRC (2007).

[bib0027] Ogunade I.M., Jiang Y., Pech Cervantes A.A., Kim D.H., Oliveira A.S., Vyas D., Weinberg Z.G., Adesogan A.T. (2018). Effects of aflatoxin B1 on performance, health, and ruminal fermentation of beef steers. Journal of Animal Science.

[bib0028] Ohkawa H., Ohishi N., Yagi K. (1979). Assay for lipid peroxides in animal tissues by thiobarbituric acid reaction. Analytical Biochemistry.

[bib0029] Pinotti L., Ottoboni M., Giromini C., Dell’Orto V., Cheli F. (2016). Mycotoxin contamination in the EU feed supply chain: A focus on cereal byproducts. Toxins.

[bib0030] Pires J.A.A., Souza A.H., Grummer R.R. (2007). Induction of hyperlipidemia by intravenous infusion of tallow emulsion causes insulin resistance in Holstein cows. Journal of Dairy Science.

[bib0031] Rushing B.R., Selim M.I. (2019). Aflatoxin B1: A review on metabolism, toxicity, occurrence in food, occupational exposure, and detoxification methods. Food and Chemical Toxicology.

[bib0032] Shi H., Zhang M., Cui Y., Chen Y., Li J., Wang Y. (2022). Growth performance, digestibility, and plasma metabolomic profiles of Saanen goats exposed to different doses of aflatoxin B1. Journal of Dairy Science.

[bib0033] Shotwell O.L., Hesseltine C.W., Stubblefield R.D., Sorenson W.G. (1966). Production of aflatoxin on rice. Applied Microbiology.

[bib0034] Sui Y., Liu Y., Guo Y., Jiang C., Li R., Qiao Y., Li J. (2022). Aflatoxin B1 exposure in sheep: Insights into hepatotoxicity based on oxidative stress, inflammatory injury, apoptosis, and gut microbiota analysis. Toxins.

[bib0035] Sunderman F.W., Nomoto S. (1970). Measurement of human serum ceruloplasmin by itsp-phenylenediamine oxidase activity. Clinical Chemistry.

[bib0036] Zhang M., Li J., Cui Y., Shi H., Chen Y., Wang Y. (2022). Evaluation of growth performance, nitrogen balance and blood metabolites of mutton sheep fed an ammonia-treated aflatoxin B1-contaminated diet. Toxins.

[bib0037] Zhao L., Zhang L., Xu Z., Liu X., Chen Y., Li S. (2022). Aflatoxin B1 exposure disrupts rumen fermentation and microbial community in dairy goats. Toxins.

